# The Delivery of Biologically Active Agents into the Nuclei of Target Cells for the Purposes of Translational Medicine

**DOI:** 10.32607/actanaturae.11049

**Published:** 2020

**Authors:** A. S. Sobolev

**Affiliations:** Institute of Gene Biology, Russian Academy of Sciences, Moscow,119334 Russia; Lomonosov Moscow State University, Moscow, 119234 Russia

**Keywords:** modular nanotransporters, polyplexes, drug delivery, antibody mimetics, gene therapy, photodynamic therapy, radiotherapy

## Abstract

Development of vehicles for the subcellular targeted delivery of biologically active agents is very promising for the purposes of translational medicine. This review summarizes the results obtained by researchers from the Laboratory of Molecular Genetics of Intracellular Transport, Institute of Gene Biology RAS, which allowed them to design the core technology: modular nanotransporters. This approach ensures high efficacy and cell specificity for different anti-cancer agents, as they are delivered into the most vulnerable subcellular compartment within the cells of interest and makes it possible for antibody mimetics to penetrate into a compartment of interest within the target cells (“diving antibodies”). Furthermore, polyplexes, complexes of polycationic block copolymers of DNA, have been developed and characterized. These complexes are efficient both *in vitro *and *in vivo *and demonstrate predominant transfection of actively dividing cells.

## INTRODUCTION

The cell nucleus, where the main program of cell function is stored, is a natural target for many biologically active substances. These substances can be divided into two large groups [1]. The first group includes agents (e.g., the cytotoxic ones) that can have a damaging effect anywhere in the cell, with the nucleus being the compartment most sensitive to them. In other words, if these agents reside in the nucleus, the same effect will be achieved at a minimal concentration compared to other localizations. The second group consists of agents that begin showing their impact from the instant they enter the cell nucleus (e.g., DNA). The present review focuses on both of these groups.

Photosensitizers (PSs) and radionuclides emitting particles with a short path length (such as emitters of alpha particles (APs) or Auger electrons (AEs)) exemplify agents from the first group. Both of them are cytotoxic agents that are widely used in medical practice to treat cancer, but they are not limited to this type of diseases. The cell nucleus is extremely sensitive to the cytotoxic agent of PSs, reactive oxygen species (i.e., singlet oxygen, hydroxyl radical, and a number of other free radicals) [2]. As for the emitters of APs and AEs, it has been known for over 50 years that the cell nucleus is the cellular compartment most sensitive to them [3]. Meanwhile, both PSs and emitters of APs/ AEs exhibit neither tropicity with respect to the cell nucleus nor cell specificity.

is little doubt that DNA needs to be delivered into the cell nucleus if its expression is to be achieved. The second group of biologically active substances also includes regulatory polypeptides whose effect mani fests upon interaction with the macromolecules of the cell nucleus.

Therefore, for the purposes of translational medicine, biologically active agents must be delivered to the nuclei of target cells so that their properties can be deployed, since most of these agents cannot reach the nuclei by themselves.

key for delivering macromolecules or other biologically active substances (for brevity, they will hereinafter be referred to as “cargo”) is to use natural intracellular transport processes, such as receptor-mediated endocytosis or nucleocytoplasmic transport (they have been described in numerous books and reviews, such as refs. [4, 5]). Accordingly, the vehicle must contain amino acid sequences or other target molecules that prompt it to move in the desired direction and to overcome the numerous barriers on its way to the nucleus (both on the target cell surface and inside it) [6].

## MODULAR NANOTRANSPORTERS

**Fig. 1:**
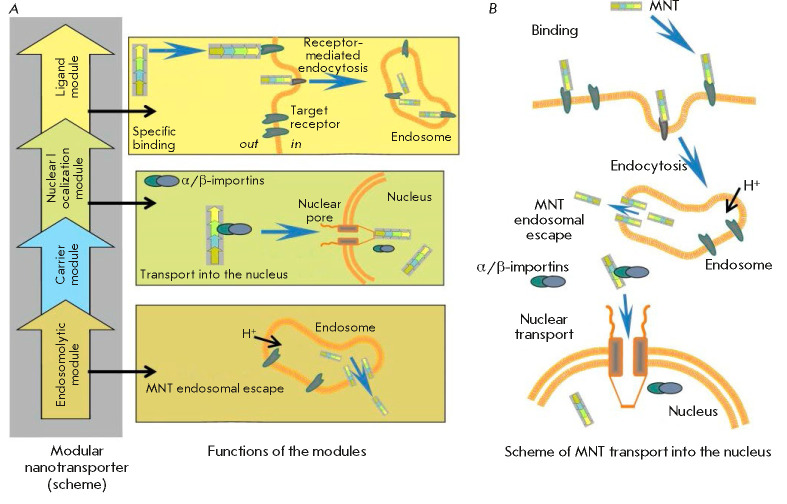
Modular nanotransporters (*A*) and the schematic representation (*B*) of how they are transported into the cell nucleus (after [15])

The modular nanotransporters (MNTs) being developed in our laboratory meet these criteria and can be regarded as a technological platform for the delivery of therapeutic agents to a given compartment of target cells of the desired type [1, 7-16]. This platform is based on: (a) use of natural processes of specific molecular recognition; (b) the previously mentioned transport inside the cell and outside of it, and (c) the principle of modularity; i.e., the ability to change the transport or recognition units/modules to adapt MNTs to the desired type of target cells, cellular compartments, the intracellular targets, and the “cargo” being delivered. A typical MNT (*Fig. 1*) consists of a ligand module, an endosomolytic module, a nuclear localization module, and a carrier module. The ligand module ensures interaction with the internalizable surface receptor and, therefore, recognition of the target cell and transport of an agent inside this cell via receptor-mediated endocytosis. The endosomolytic module has the function of pH-dependent pore formation in the endosomes, and thus it ensures release of the MNT, with the active component being delivered from these compartments with weakly acidic contents to the target cell cytosol. The nuclear localization module contains an amino acid sequence that acts as a nuclear localization signal (NLS) specifically interacting with the importin complex in the cytosol and ensuring transport of the agent through the nuclear pore. The carrier module is used to join other modules into an integral whole and attach the “cargo.” Along with the aforementioned four modules, the MNT can also contain other modules if interaction with some additional intra- and extracellular components is required. Thus far, the properties of the following MNTs are the ones that have been studied most thoroughly both *in vitro *and *in vivo*:

•MNTs with epidermal growth factor (EGF) as a ligand module (MHT_EGF_), which exhibit specificity with respect to cells (Examples: cells of bladder cancer, head and neck cancer, glioblastoma, and colorectal cancer [17, 18]) overexpressing epidermal growth factor receptors (EGFR) [9, 19, 20],

•MNTs with the α-melanocyte-stimulating hormone as a ligand module (MHT_MSH_), which exhibit specificity with respect to cells (An example: melanoma cells [21]) overexpressing melanocortin-1 receptors [8, 12, 22],

•MNTs with folic acid as a ligand module (MHT_F_) targeted at cells (Examples: cervical and ovarian cancer cells [10]) overexpressing folate receptors [23, 24].

The compositions of the modules of these MNTs are as follows:

MNT_EGF_: DTox-HMP-NLS-EGF;

MNT_MSH_: DTox-HMP-NLS-αMSH;

MNT_F_: DTox-HMP-NLS-PEG-FA,

where DTox is a fragment of the translocation domain of the diphtheria toxin (the endosomolytic module); HMP is the hemoglobin-like protein of *E. coli *(the carrier module); NLS is the optimized nuclear localization sequence of the SV40 large T antigen (the nuclear localization module); EGF, αMSH, and FA are epidermal growth factor, melanocyte-stimulating hormone, and folic acid, respectively (the ligand modules); and PEG is bifunctional polyethylene glycol.

Other MNT variants have been developed or are currently being developed. They are discussed in the sections below.

When fragments of molecules join together to form a single molecule (as is the case when creating an MNT), it is not obvious that the resulting chimeric molecule will retain the properties of these fragments. Thus, its domains may spatially mask one another and impede the interactions with cellular proteins that are required for the functioning of the chimeric molecule, so the designed artificial molecule will not have the purposive properties. Both the structure of MNTs and the ability of their modules to perform their functions were studied to test the performance of MNTs.

According to dynamic light scattering data, the dimensions of MNT_MSH_ and MNT_EGF_ are 8.3 ± 0.6 and 10.6 ± 0.5 nm [19], respectively.

Numerous attempts to crystallize MNTs for further study of their structure by high-resolution X-ray diffraction analysis have failed. However, small-angle X-ray scattering, atomic force and electron microscopy [25] have shed some light on the structures of MNT_MSH_ and MNT_EGF_. An important conclusion drawn from this structural study is that the endosomolytic and the ligand modules are spatially separated sufficiently well. Their mutual masking and the loss of their functions are, therefore, eliminated.

This conclusion was convincingly confirmed by tests performed to evaluate the performance of the MNT modules of all developed types. To save space, let us just provide few examples. Thus, the dissociation constant (*K*_d_) of the MNT_EGF_-EGFR complexes was 29 nM, which is close to that of the EGF-EGFR complexes [9]. For the complexes formed between MNT_MSH_ and melanocortin receptors, *K*d was approximately 20 nM [8]. The studied MNTs exhibited a membranolytic activity in two pH ranges: at pH 5.5–6.5 (the range being close to that of endosomes and mediated by DTox) and at pH range of 3–4, which is caused by the action of HMP [8, 9]. The membrane pores created by the MNTs have been characterized electrochemically and by atomic force microscopy [7, 9, 22]. After the full-length MNT_MSH_ (i.e., the ones containing all four modules) had been added to the planar lipid bilayer at pH 5.5, ion channels with a conductivity of ~ 2–5 nS appeared. Meanwhile, MNT_MSH_ without the endosomolytic module did not form ion channels at pH 5.5. The channels did not appear even under the action of full-length MNTs at a neutral pH (7.0), thus proving that the endosomolytic module exhibits its membrane activity in acidified milieus. Five to fifteen minutes after the milieu had been acidified to pH 5.5, MNT_EGF_ formed ring structures 30–50 nm in diameter in the lipid bilayer, as detected by atomic force microscopy. Fluctuating holes 50–200 nm in diameter permeating the lipid bilayer could be detected after 40–60 min. The function of the endosomolytic module was also demonstrated in living cells (Cloudman S91 mouse melanoma, the M3 clone) [8] by measuring the pH of the intracellular microenvironment of MNT_MSH_ by fluorescence ratio image microscopy. The MNT_MSH_ without the endosomolytic module (DTox) resided in vesicles with weakly acidic and acidic contents, while the full-length MNT_MSH_ (with the DTox module) was located in the neutral microenvironment. This result demonstrates that the full-length MNT_MSH_ can escape from the acidified endocytic compartments of living cells. The interaction between the NLS-carrying module within various MNTs and α/β-importin dimers ensuring the delivery of NLS-carrying proteins to the cell nucleus has been characterized by several methods (surface plasmon resonance and thermophoresis) [9, 26]. The measured constants of the affinity of MNTs to importin dimers were close to that of a free natural polypeptide carrying the same NLS. Hence, it was demonstrated that all the modules within a chimeric artificial MNT molecule had retained their functions.

**Fig. 2:**
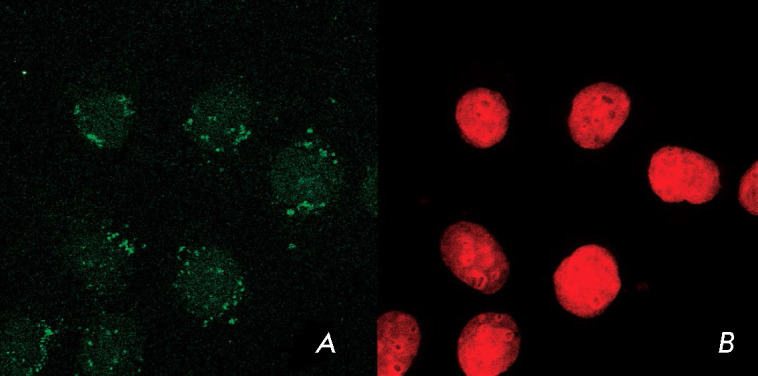
Subcellular MNTEGF localization within A431 human epidermoid carcinoma cells (after [9] with permission). The A431 cells were incubated for 4 h with MNTEGF in a culture medium, then washed and incubated in the medium without MNTEGF. (*A*) – immunocytochemical detection of MNTEGF, (*B*) – nuclear DNA detection with ToPro-3 in the same group of A431 cells

Therefore, all the full-length MNTs penetrated the target cells via receptor-mediated endocytosis (as confirmed by the fact that the specific ligands of the respective receptors inhibited their penetration) and localized within their cell nuclei [8, 9, 14, 23, 26, 27, 28] (*Fig. 2*), as was actually planned by the authors in order to solve the problem related to “cargo” delivery into the nucleus.

Indeed, PSs such as the chlorin *e*6 and bacteriochlorin *p *attached to MNT_EGF_ or MNT_MSH_ are hundreds and thousands of time more cytotoxic than the free ones. Thus, in the experiments on A431 cells overexpressing EGFR, the half-maximal effective concentration (*EC*_50_) of MNT_EGF_-chlorin *e*6 was 0.53 nM, while *EC*50 of free chlorin *e*6 was 1780 nM (i.e., 3,360-fold higher [9]). In other words, the same cytotoxic effect of the chlorin *e*6 photosensitizer can be achieved by using concentrations 3,360 times as low by moving this photosensitizer to the nucleus using MNTs. The same experiments demonstrated that MNT_EGF_ made the PS specific to certain cells. Thus, whereas free chlorin *e*6 was cytotoxic against both the target A431 cells and non-target NIH 3T3 fibroblasts lacking EGFR, MNT_EGF_-chlorin *e*6 affected the target cells only.

Qualitatively similar results were obtained in the experiments with MNT_MSH_ [8], where it was also demonstrated that an MNT must contain all four modules. Thus, an MNT lacking the endosomolytic module was 5.3 times less active than the full-length MNT, while the MNT lacking the NLS-containing module was even less cytotoxic.

It has been convincingly demonstrated in *in vivo *experiments on tumor-carrying mice that PSs are efficiently delivered to the nuclei of cancer cells using MNTs [19, 31]. An immunocytochemical analysis of the distribution of MNT_EGF_ and MNT_MSH_ injected intravenously to tumor-carrying mice revealed that MNTs preferentially accumulate in cancer cells (to be more specific, in the nuclei of cancer cells). The experiment involving intravenous injection of MNT_MSH_ for the treatment of experimentally induced melanomas showed that photosensitizer bacteriochlorin *p *delivered by MNT_MSH_ inhibits B16-F1 tumor growth 85–89% more efficiently compared to free bacteriochlorin *p*; the inhibition of the growth of Cloudman S90 melanoma was 93% more efficient. The ratio between the PS concentrations in the tumor and in the skin was as high as 9.8, some 4.5 times higher than that observed for free bacteriochlorin *p *[32]. A significant therapeutic effect was also uncovered for an intravenous injection of MNT_EGF_ with photosensitizer chlorin *e*6 in a model of A431 human epidermoid carcinoma grafted into immunodeficient mice: 75% of the mice survived by day 92, while only 20% of the mice treated with free chlorin *e*6 (positive control) and none of the untreated animals survived by day 23.

APs and AEs cause dense ionization and thus efficiently damage the molecules along their tracks; the path length of these particles in tissues is rather short: 50–100 µm (i.e., several cell diameters) for APs and several dozens or hundreds nanometers for AEs (i.e., they are almost equal to the dimensions of the cell nucleus). These features of emitters of APs and AEs are rather attractive owing to the fact that in the case when they are selectively delivered to the target cells, one can expect the damage to the surrounding normal cells to be minimal. Meanwhile, both types of radiation cause multiple double-strand DNA breaks that are hardly repairable. In fact, nuclear DNA is the main target of the cytotoxic activity of these radiation types [33, 34]. Their cytotoxicity practically does not drop as the oxygen content decreases [35] (the so-called “oxygen effect” that is characteristic of sparsely ionizing radiation), so that these types of radiation have a special advantage in damaging hypoxic cancer cells. Emitters of AEs are also quite interesting, because up to several dozen AEs are produced per decay (depending on the nature of the emitter), thus ensuring a high biological efficiency for these species if their decay occurs in close proximity to DNA [36]. Taking into account the aforementioned features, APs and, especially, AEs are of interest for the treatment of malignant tumors located in such places where damage to the surrounding normal tissues must be minimized (e.g., brain tumors, especially in children) [37], or for the treatment of micrometastases [38].

The α-particle emitter 211At, which has been used as a source of APs in experiments with MNTs, is considered one of the most promising radionuclides for therapeutic purposes [30]. The AP emitter 211At has a relatively short half-life (7.2 hrs); the path length of APs emitted by it can reach up to 70 µm; the resulting yield of double-strand DNA breaks is rather high [39]. In experiments with A431 human epidermoid carcinoma cells, as well as two human glioblastoma lines (D247MG and U87MG.wtEGFR), the cytotoxicity exhibited by 211At-MNT_EGF_ was 8- to 18-fold higher than that of 211At not delivered to the nuclei of these cells [40]. It also turned out that delivery of this emitter of APs to the cell nucleus enabled the effects of recoil nuclei, which are not revealed for other intracellular localizations because of their extremely short path length.

The following emitters of AEs, which are widely used in medicine as sources of gamma radiation, were employed in the experiments with MNTs: 125I, 67Ga, and 111In. On average, they emit 24.9, 4.7, and 14.7 AEs per decay, respectively [41]. The yield of double-strand DNA breaks caused by AEs significantly depends on the distance between a DNA molecule and the emitter of AEs [42].

^125^I or 67^Ga^ delivered by MNT_EGF_ accumulated rather intensively in the nuclei of A431 human epidermoid carcinoma cells [27, 28]: by the first hour of incubation, about 60% of all the radioactivity pumped into the cells was found in their nuclei. 125I-MNT_EGF_ was 3,500 times more cytotoxic to A431 cells than the 125I-iodinated control polypeptide, which had not penetrated the cells [27]. Similar results were obtained for 67Ga [28] and 111In [20]: the cytotoxicity of the emitters of AEs delivered to the cell nuclei increased abruptly. In these experiments conducted for three cell lines (A431, D247MG, and U87MG.wtEGFR), the cytotoxicities of 125I and 67Ga delivered into the cell nucleus by MNTs were compared to those of the radionuclides delivered mostly into the cytoplasm. As might be expected, the delivery of these emitters into the nucleus ensured a significantly higher cytotoxicity (20- to 400-fold depending on the particular radionuclide and cell line) [15].

Safety testing of MNTs during preclinical studies conducted at the National Medical Research Radiological Center of the Ministry of Health of the Russian Federation showed that the studied MNTs injected intratumorally exhibited a very low toxicity (both acute and chronic) in mice and rats, low immunogenicity/ allergenicity in mice and guinea pigs, and were not pyrogenic in rabbits [19, 43, 44, 45]. In general, this therapeutic approach, involving intratumoral injection of MNTs, was considered safe [46].

**Fig. 3:**
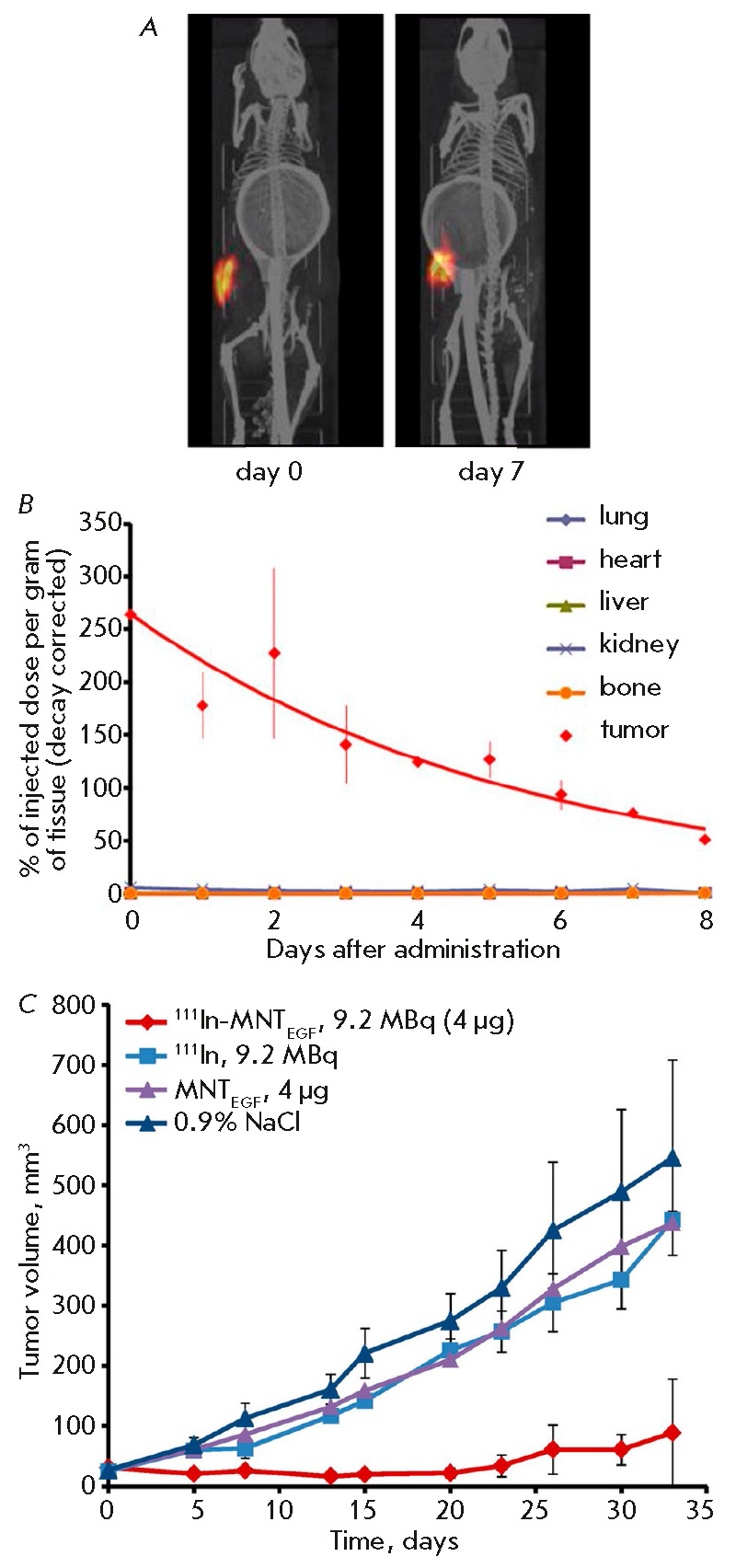
Administration of 111In-MNT_EGF_ into subcutaneous tumors (EJ human bladder cancer) transplanted to immunodeficient Balb/c *nu/nu *mice (after [20] with changes): (*A*) – SPECT/CT visualization of radioactivity retention within the tumor; (*B*) – the kinetics of radioactivity retention by the tumor and normal tissues; (*C*) – antitumor efficiency of 111In-MNT_EGF_ after intratumoral administration

^111^In-MNT_EGF_ administered as a single dose into human bladder carcinoma (EJ) grafted subcutaneously to immunodeficient Balb/c *nu/nu *mice was retained inside the tumor for a rather long time (its retention half-time in the tumor was 4.1 ± 0.5 days) [20]; no more than 0.5% of the injected dose entered the blood. When delivered intratumorally, 111In-MNT_EGF_ exhibited a pronounced dose-dependent therapeutic effect on EJ tumors (up to 90% compared to the untreated control (both non-labeled MNT_EGF_ and free 111In) at the same dose) [20] (*Fig. 3*).

Another variant of MNTs, 111In-MNTF, exhibited a similar therapeutic effect [23, 24]. 111In-MNTF ensured a dose-dependent growth inhibition of subcutaneously grafted tumors (cervical cancer HeLa cells) in immunodeficient mice (up to 80%); the survival rate of the animals was as high as 60% (by day 90), while all the untreated animals in the control group died by day 21.

The results obtained using different cytotoxic agents (two PSs, one emitter of APs, and three emitters of AEs) have motivated researchers to view MNTs as prospective agents for the delivery of a much wider range of biologically active molecules. In this sense, bioactive polypeptides are particularly attractive because MNTs are actually chimeric polypeptides and inclusion of additional polypeptide fragments into their composition is a problem that can be solved using genetic engineering methods.

The MNT carrying a fragment of the p21 protein, p21-MNT_EGF_, is one of the variants of such MNTs. The p21 protein exhibits a broad range of activities: it affects DNA repair and controls the DNA replication fork by forming a complex with the PCNA protein, and it regulates the cell cycle by interacting with cyclins and cyclin-dependent kinases [47]. This makes p21 or its fragments through which it binds to PCNA an attractive tool for modifying the action of DNA-damaging agents (e.g., those used in cancer therapy). The p21-MNT_EGF_ that contained the C-terminal fragment of protein p21 (amino acid residues 87–164), with the site through which p21 binds to PCNA, was synthesized based on these starting points [48]. DNA was damaged by bleomycin, an anticancer drug that causes double-strand DNA breaks [49]. The comet assay in an alkaline medium, which allows one to detect all types of DNA breaks, was used to analyze and repair damage to DNA. Pre-incubation of A431 cells with p21-MNTEGF showed that p21-MNT_EGF_ statistically significantly inhibits DNA repair compared to the control, MNT_EGF_ (i.e., similar MNTs not carrying the p21 fragment) [14].

The encouraging results of this study have contributed to further progress towards the targeted intracellular delivery of biologically active polypeptides. MNTs carrying an antibody mimetic anti-Keap1 monobody (which activates the Nrf2/ARE signaling pathway through competition with endogenous Nrf2 for binding to the Keap1-inhibiting protein) as the effector part were designed under the Russian Science Foundation Grant No. 17-14-01304 [50]. The transcription factor Nrf2 regulates several hundred genes (some of them involved in cell defense against oxidative stress (i.e., antioxidant defense), while others participate in the defense against toxic xenobiotics and a number of other vital processes) [51]. Oxidative stress accompanies or is involved in the pathogenesis of many diseases, such as Parkinson’s disease, Huntington’s disease, diabetes mellitus, atherosclerosis, cell senescence, radiation-induced cell damage, etc. [51, 52]. In the absence of oxidizing agents, Nrf2, which forms a complex with its inhibitor Keap1 in the cytoplasm, undergoes ubiquitination, followed by degradation in proteasomes. The xenobiotic oxidizing agents appearing in the cell interact with the thiol groups of “cysteine sensors” within Keap1, which leads to Nrf2 release and accumulation in the nucleus, followed by its interaction with the “antioxidant response element” (ARE) within the domain of the promoters of controllable genes, thus activating their transcription [51, 53]. The experiments with MNTs containing the anti-Keap1 monobody revealed a statistically significant increase in the expression level of a number of antioxidant defense genes. Furthermore, the cells were protected against the oxidative stress induced by *tert*-butyl hydroperoxide. It was shown for the mouse model of oxidative stress induced by hepatotoxin acetaminophen that the preliminary administration of MNTs with anti-Keap1 monobody activating the Nrf2/ARE signaling pathway inhibits the hepatotoxic action of the acetaminophen that is detected according to elevated serum aspartate aminotransferase and alanine aminotransferase activity [50]. These results indicate that MNTs can be used to deliver antibody mimetics both *in vitro *and *in vivo*.

## POLYPLEXES (COMPLEXES OF CATIONIC BLOCK COPOLYMERS OF DNA) FOR DELIVERING GENETIC MATERIAL

For decades, the potential opportunity to change the function of cells by modifying their genetic program has been stimulating researchers who focus on gene therapy (e.g., for treating cancer or hereditary diseases) or bioengineering for the production of the target macromolecules, etc. As often happens when trying to solve such important problems consisting of several large tasks, finding the optimal solution to one of them is far from obvious. Targeted delivery of genetic material is one such task. A natural solution to this problem could involve viruses, as they are the supramolecular structures best suited for overcoming the barriers at the organism and cellular levels during targeted delivery of virus’s own genetic material. The largest number of gene therapy preclinical and clinical studies has centered on these viruses. However, viral vectors are associated with a risk of unexpected, and often severe, adverse events, which will actually remain a problem for quite a long time [54, 55]. Therefore, simultaneously with the design of viral vectors for the delivery of genetic material, non-viral methods that arouse increasing interest among researchers are currently under development.

One of the variants of non-viral delivery is to use polycations, which form complexes with the nucleic acids known as polyplexes. In polyplexes, DNA (or RNA) is packaged and protected against hydrolytic enzymes, so that these complexes remain sufficiently stable in biological environments. Polyplexes are non-pathogenic. Many of them are also non-immunogenic and low-toxic. By modifying the original polymers, one can obtain particles with different properties, as well as attach different functional components to impart such properties as cellular specificity or other tailored properties to the complexes [56].

It is obvious that in order to achieve these favorable properties, the polymeric vehicles within the polyplexes need to be supplemented with the aforementioned functional components. Furthermore, the polymer composition also needs to be optimized to bring the properties of the polyplexes closer to those of virions capable of delivering genetic material.

Let us consider the example of the well-known polyethyleneimine (PEI)-based polyplexes. Particles of different sizes and charges form when PEI is mixed with DNA in different proportions (expressed as the N/P ratio, where N is the number of amino groups of PEI and P is the number of DNA phosphate groups). To increase the time during which the polyplexes circulate in the blood and to reduce the toxicity of PEI, PEG is attached to PEI, yielding PEG-PEI block copolymers. Since both the N/P and PEG/PEI ratios can be variegated, the resulting problem to be solved involves finding the optimal ratio between the components in the polyplex. To solve this problem, polyplex variants with different ratios between the components were tested on 11 cell lines; transfection efficiency was assessed according to the activity of the expressed reporter gene [57]. It was discovered that the resulting dependences of transfection efficiency on the N/P and PEG/PEI ratios were non-monotonous, but that their shapes were similar for all the analyzed cells. Furthermore, importantly, maximum transfection efficiencies for different cell lines were observed at the same N/P and PEG/PEI ratios. A significant, positive correlation between the transfection efficiency and the percentage of nanoparticles within polyplexes sized 50–75 nm was revealed for all the investigated cell lines. This result, obtained for more than 10 human and animal cell lines, allows one to transfect different cell lines with maximum efficiency. However, whereas the dependences of transfection efficiency on the N/P and PEG/PEI ratios were similar, there was also a significant difference for all the analyzed cell lines: the maximum achievable transfection efficiency varied from almost 100% (HeLa, HEK293, Cloudman melanoma, and B16-F1 melanoma) to 4.4% (BT-474 cells). These differences could be attributed either to the differences in reporter gene expression or to the differences in the transport and unpacking of polyplexes observed across the cell lines. Experimental testing [57] showed that the second assumption was true: the transfection efficiency showed a positive correlation with the rate of polyplex entry into the cells and a negative correlation with the rate of their unpacking in the endocytic compartments.

Modifying block copolymers with ligands specific to internalizable receptors on the target cells impart cellular specificity to the polyplexes. Thus, the polyplexes containing αMSH acquired specificity with respect to melanoma cells overexpressing melanocortin 1 receptors (αMSH is their ligand) and showed a much greater efficiency in *in vivo *transfection of these cancer cells [58].

**Fig. 4:**
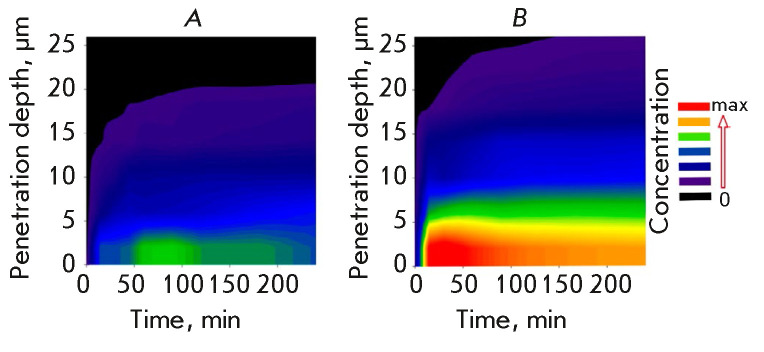
Polyplex penetration into the tumor (after [62] with permission). (*A*) – control; (*B*) – after inhibition of collagen I production with losartan

The size of PEI-based polyplexes ensuring the most efficient transfection (50–75 nm; see the text above) casts doubt on whether nanoparticles of this size can penetrate through nuclear pores into the nucleus of a non-dividing cell, because the known size limit is ap proximately 40 nm even for NLS-carrying particles [59]. The experiments on transfection of cells fluorescently labeled with polyplexes showed that ~ 90% of the cells expressing the reporter gene delivered by these nanoparticles had been transfected during the cell division [60]. Therefore, it is possible to regard polyplexes as a means suitable for the transfection of actively dividing cells (first of all, the cancer ones). The average number of intact DNA molecules per nucleus of a successfully transfected cell was also estimated in this study [60]. It was found to be equal to ~ 3, which indicates that the transfection efficiency of the polyplexes was rather high. The physical properties of polyplexes also suggest that it is reasonable to use them in cancer gene therapy: thus, cancer tumors (or, to be more precise, their vessels), exhibit the so-called effect of “enhanced permeability and retention” of nanoparticles [61]. PEI-based polyplexes modified with αMSH showed different levels of efficiency in the transfection of B16-F1 and Cloudman S91 melanoma cells: the transfection efficiency was higher for B16-F1 melanoma cells compared to that for Cloudman S91 melanoma cells. As it has been shown, the reason for these differences is that B16-F1 tumors are more vascularized and their endothelium is more likely to be fenestrated, which makes the “enhanced permeability and retention effect” more pronounced [62]. Nevertheless, tumor tissues act as a barrier for polyplex nanoparticles. Although these nanoparticles penetrate tumor tissues unlike normal ones, the penetration depth is rather small (≤ 20 µm) [63] (*Fig. 4A*). Therefore, if polyplexes need to be delivered into a tumor to a greater depth, there should be some additional impact on the tumor. One of the variants allowing one to increase both the penetration depth of polyplexes and their concentration in the tumor is to modify the tumor interstitium (e.g., by inhibiting the production of collagen type I) [62, 64] (*Fig. 4B*).

PEI-based polyplexes have shown therapeutic efficacy in the case of experimentally induced tumors (S37 mouse sarcoma [65] and Cloudman S91 melanoma, clone M3 [58]). In earlier experiments involving polyplex-based mammary gland transfection in mice and sheep, the target protein was produced with their milk [66]. The same polyplexes could be used for transgenosis of early mouse and rabbit embryos [67].

## CONCLUSIONS

Having summed up the results of the studies conducted on this topic, the following conclusions can be drawn.

(A) Regarding the delivery of cytotoxic agents using modular nanotransporters for cancer therapy: Modular nanotransporters (a technological platform, i.e., the core technology that serves as the basis for solving particular tasks) have been developed. This technology makes it possible to impart cellular specificity and high efficiency to a large number of antitumor agents by delivering them to the cell nucleus using the natural processes of intracellular transport.

(B) Regarding the delivery of biologically active polypeptides: Modular nanotransporters have been used to design antibody mimetics (the so-called “diving antibodies”) capable of penetrating living cells and affecting the function of target molecules; furthermore, a new type of modular nanotransporters that affect the functions of transcription factors in cells both *in vitro *and *in vivo *has been designed. We believe that the approach being currently developed can lead to a breakthrough in the design of tools for the study of the function of living cells and, possibly, in the development of therapeutic agents.

(C) Regarding the delivery of genetic material using polyplexes: It has been demonstrated that polyplexes preferentially transfect dividing cells, which should be taken into account during the potential practical use of polyplexes. The efficiency of transfection using polyplexes has been demonstrated both *in vitro *and *in vivo*.

The hope is that the range of biologically active agents delivered into the cell (first of all, antibody mimetics) will be subsequently broadened: novel “diving antibodies” could be designed, and humanized MNTs for potential systemic use could be obtained. These studies have already started [68, 69].

## Abbreviations

AE: Auger electron
αMSH: α-melanocyte-stimulating hormone
AP: alpha particle
ARE: antioxidant response element
DTox: a fragment of the diphtheria toxin translocation domain
EC50: half maximal effective concentration
EGF: epidermal growth factor
EGFR: EGF receptor
FA: folic acid
FR: FA receptor
HMP: hemoglobin-like protein of E. coli
Kd: dissociation constant
MNT: modular nanotransporters
MNTEGF: MNT with EGF as a ligand module
MNTF: MNT with folic acid as a ligand module
MNTMSH: MNT with αMSH as a ligand module
NLS: nuclear localization signal
Nrf2: transcription factor regulating, in particular, expression of antioxidant response genes
PEG: polyethylene glycol
PEI: polyethyleneimine
PS: photosensitizer.
